# The association between the incident risk of Parkinson’s disease and depression in middle-aged and older adults, and the moderating role of lifestyle: evidence from the CHARLS

**DOI:** 10.3389/fpsyg.2025.1590931

**Published:** 2025-06-18

**Authors:** Wu-xiao Wei, Lian Meng, Zhen-fang Mao, Zhen-hua Mo, Lu Yang, Yan Qin, Jin-yu Huang

**Affiliations:** ^1^Guangxi University of Science and Technology First Affiliated Hospital, Liuzhou, China; ^2^Youjiang Medical University for Nationalities, Baise, China

**Keywords:** Parkinson’s disease, depression, lifestyle, risk factors, CHARLS, China

## Abstract

**Background:**

Parkinson’s disease (PD) and depression frequently coexist in middle-aged and older populations, potentially influencing each other. This study explores the relationship between depression and PD risk and investigates the association between lifestyle score and PD risk.

**Methods:**

Data from the China Health and Retirement Longitudinal Study (CHARLS) for adults aged 45 and above were analyzed. A total of 30,347 participants were included, with 565 individuals developing PD during follow-up. PD cases were self-reported physician diagnoses. Depression was assessed using the CESD-10 scale (score ≥ 10 indicating depression). Lifestyle factors (smoking, drinking, social activity, sleep, and BMI) were scored as healthy (≥4) or unhealthy (<4). Cox proportional hazards models were used to analyze PD risk, and cubic spline regression was employed to evaluate the dose–response relationship between depression, lifestyle, and PD risk.

**Results:**

Depression (CESD-10 ≥ 10) was significantly associated with an increased risk of PD. In the fully adjusted model (Model 4), individuals with depression had a 53% higher risk of developing PD compared to those without depression (HR = 1.53, 95% CI: 1.28–1.83). Cubic spline regression revealed a dose–response relationship: as CESD-10 scores increased, the risk of PD also increased. Unhealthy lifestyle was significantly associated with a higher risk of PD. The analysis showed that individuals with an unhealthy lifestyle had a 23.5% higher risk of developing PD than those with a healthy lifestyle. Additionally, the risk of PD varied with different lifestyle components. For example, no-smoking had a 17.9% lower risk of developing PD compared to smoking, and individuals with long sleep durations had a 36.2% lower risk of PD compared to those with short sleep durations.

**Conclusion:**

Depression is significantly associated with the risk of PD in middle-aged and older populations. Our findings show a strong link between an unhealthy lifestyle and PD risk. This highlights the importance of addressing depression and avoiding unhealthy lifestyles in PD prevention.

## 1 Introduction

Parkinson’s disease (PD) is the second most common neurodegenerative disease, with its prevalence expected to double over the next 30 years (Tolosa et al., 2021). Studies have shown that the incident of PD is closely related to age, with a higher incidence in individuals over the age of 60, and a higher incidence in men compared to women (Van Den Eeden et al., 2003; Kalia and Lang, 2015). According to epidemiological studies, the prevalence of PD in North America and Europe ranges from 100 to 300 cases per 100,000 people (Pringsheim et al., 2014). Additionally, some studies suggest that as the population in these regions ages, the incidence is rising in Asian countries, such as China and India (Kalia and Lang, 2015), presenting new challenges for public health systems and healthcare resources.

PD is primarily characterized by motor symptoms, such as tremors, rigidity, and bradykinesia (Kalia and Lang, 2015; Jankovic, 2008). In addition, PD also involves non-motor symptoms, including depression, which are widespread and significantly affect the quality of life of patients. These symptoms are often considered core features of the disease and may appear even before motor symptoms emerge (Poewe et al., 2017; De Lau and Breteler, 2006; Cong et al., 2022; Lian et al., 2019). Depression is highly prevalent in PD patients, with an estimated 40% of individuals experiencing clinically significant depressive symptoms (Reijnders et al., 2008). Although depression has been widely studied as a risk factor for PD (Wang et al., 2018; Jeong et al., 2021), the role of lifestyle factors in moderating this relationship remains insufficiently explored. This study uniquely examines how a lifestyle score, encompassing factors such as smoking, drinking, sleep, and social activities, may mitigate the association between depression and PD risk. By incorporating lifestyle as a moderating factor, this research provides a novel approach to understanding and potentially preventing the onset of PD in middle-aged and older adults. While previous studies have shown that lifestyle factors, such as smoking, drinking, social activity, and sleep, influence the risk of PD (Jacobs et al., 2020; Paul et al., 2019; Marinus et al., 2018; Ascherio and Schwarzschild, 2016; Cusso et al., 2016), there are significant regional and national differences in the research progress on the relationship between PD and depression, as well as the strategies for lifestyle interventions. These differences significantly increase the burden on patients and their families (Alshimemeri et al., 2024), thus highlighting the urgent need for further investigation into the relationship between lifestyle and PD.

In developed countries, such as those in Europe and the U. S., the relationship between PD and depression has been widely studied, and various factors have been found to be closely related to the onset and progression of both conditions. One study found that the risk of developing PD was 2.2 times higher in individuals with depression compared to those without (Wang et al., 2018). PD is also closely related to gender, education level, disease duration, severity, genetic factors, and non-motor symptoms. Research by Cong et al. revealed significant effects of these factors on the occurrence of depression (Cong et al., 2022). Lifestyle factors, such as smoking and sleep duration, have an important impact on the risk of PD, and appropriate lifestyle interventions may help slow the progression of the disease (Ascherio and Schwarzschild, 2016; Reichmann et al., 2022). In contrast, in Asian countries, factors such as depression, culture, and society have a more significant impact on the risk of PD. For example, studies in Japan have shown that 38% of Parkinson’s patients exhibit higher levels of depression, partly due to cultural influences on mental health issues (Inoue et al., 2010). Jeong et al.’s study found that the risk of PD significantly increased in elderly Koreans as the severity of depression increased, and appropriate management of depression could reduce the risk of PD (Jeong et al., 2021). Cui et al. found that the prevalence of depression in Chinese Parkinson’s patients was 11.17%, with lifestyle factors such as smoking, drinking, social activity, daily living activities, and sleep quality being associated with the condition (Cui et al., 2017; Xu et al., 2025).

Our data comes from the China Health and Retirement Longitudinal Study (CHARLS), a large-scale study of middle-aged and elderly populations, providing a unique opportunity to explore the interactions between depression, lifestyle factors, and PD. Currently, there is insufficient research on the relationship between the incident risk of PD and depression in middle-aged and elderly populations, and the moderating role of lifestyle factors has not been adequately analyzed. Therefore, further research is necessary to provide more targeted and personalized intervention strategies for patients globally.

## 2 Methods

### 2.1 Study population

This study uses data from the CHARLS, a nationally representative longitudinal cohort study that started in 2011. It covers 150 counties and 450 villages across 28 provinces in China, with over 17,000 participants and 10,000 households. Follow-up surveys are conducted every 2–3 years, collecting demographic, lifestyle, and health data through face-to-face interviews. CHARLS has been approved by the Biomedical Ethics Committee of Peking University, and participants provided informed consent. Data is accessible via the official website.^1^

**Figure 1:**
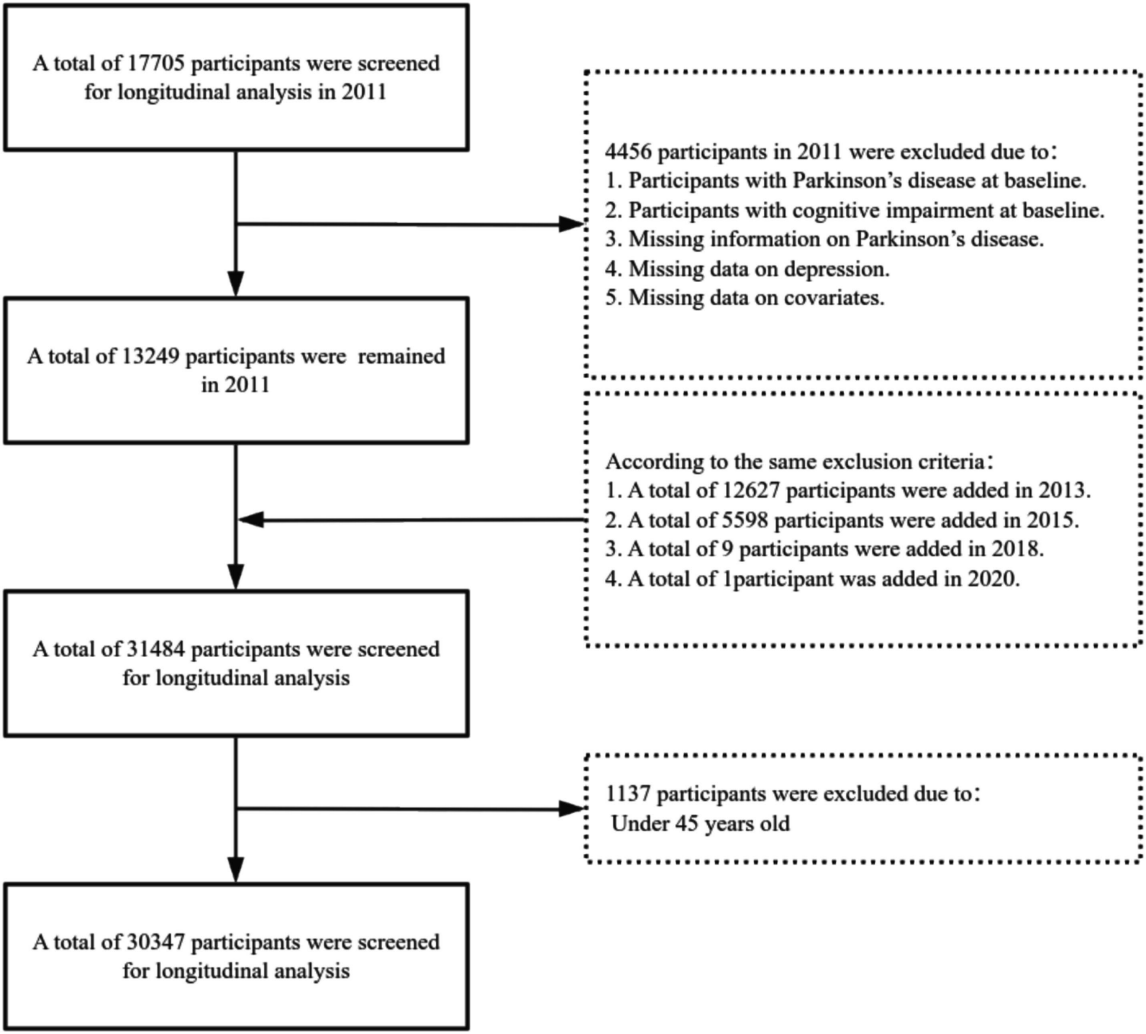
Flowchart showing the selection of participants into the final analysis of this study.

This study retrospectively analyzed the data collected from CHARLS in 2011, 2013, 2015, 2018, and 2020, focusing on participants aged 45 and above. The aim was to explore the relationship between the incident risk of PD in middle-aged and older adults and to analyze the moderating role of a lifestyle in this relationship. To ensure data quality and consistency, participants were excluded based on the following criteria: (1) those already diagnosed with PD at baseline, (2) participants with missing data on depression, (3) participants with missing data on PD, (4) participants with incomplete covariate information, (5) those with cognitive impairment at baseline, and (6) participants under the age of 45. Ultimately, 30,347 eligible samples were included for analysis. The specific sample selection process is shown in Figure 1.

### 2.2 PD definition

PD cases were determined through self-reported physician diagnoses during follow-up. The participants were queried about whether they had received a formal PD diagnosis; If so, the corresponding participants were considered to have PD; if not, they were considered not to have PD.

### 2.3 Depression definition

Participants’ depression was assessed using the 10-item short version of the Center for Epidemiological Studies Depression Scale (CESD-10), which has demonstrated high validity and good psychometric properties among older adults in China (Andresen et al., 1994; Zhou et al., 2021). The CESD-10 consists of 10 items, each offering four response options: (1) “Rarely or none of the time” (<1 day), scored as 0; (2) “Some or few times” (1–2 days), scored as 1; (3) “Occasionally or a moderate amount of times” (3–4 days), scored as 2; and (4) “Most or all of the time” (5–7 days), scored as 3. The total score can range from 0 to 30, with lower scores indicating a lower level of depression. Individuals with a CESD-10 score of 10 or higher were classified as having depression, while those with scores below 10 were classified as not having depression.

### 2.4 Lifestyle score definition

The lifestyle score was constructed based on the following five factors: smoking, drinking, social activity, sleep duration, and BMI (Liu et al., 2024). Each factor was scored as “yes” (1 point) or “no” (0 points). Specifically: (1) Smoking: If the participant had never smoked, they received 1 point for smoking status; otherwise, they received 0 points. (2) Drinking: If the participant had not consumed alcohol in the past year, they received 1 point for alcohol status; otherwise, they received 0 points. (3) Social Activity: If the participant had engaged in any social activity in the past month, they received 1 point; otherwise, they received 0 points. Social activities included interacting with friends, playing mahjong, chess, or cards, joining a community club, engaging in sports, participating in social organizations, clubs, community-related organizations, volunteering or charitable work, and attending educational or training courses. (4) Sleep Duration: If the participant slept more than 6 h per night, they received 1 point for sleep duration; otherwise, they received 0 points. (5) BMI: This was measured by standard methods, and participants with a BMI of ≥18.5 kg/m^2^ received 1 point; otherwise, they received 0 points. The scores for these five factors were combined to create a composite lifestyle score, ranging from 0 to 5. In this study, lifestyle was classified as “healthy” (total score ≥ 4) and “unhealthy” (total score < 4).

### 2.5 Other clinical characteristics

Sociodemographic variables included age, gender, education level, marital status, residence, and personal income level. Education level was classified as: primary school or below, middle school, and high school or above. Marital status was classified as married, divorced/widowed/separated, or single. Personal income level was categorized as <10,000 RMB or ≥10,000 RMB. Lifestyle variables included smoking, drinking, sleep duration, BMI, and social activity. Sleep duration was classified as less than 6 h/day or more than 6 h/day.

### 2.6 Statistical methods

Descriptive statistical analysis was conducted to summarize sample characteristics, and chi-square tests were used to assess differences among categorical variables. The association between depression and the risk of PD onset was analyzed using Cox proportional hazards regression models, with sequential adjustments for sociodemographic, lifestyle, and health-related variables. Additionally, sensitivity analyses were performed to verify the robustness of the results, and a cubic spline model was used to explore the nonlinear relationship between lifestyle and the risk of PD onset. All statistical analyses were conducted using Stata 16.0. A two-tailed significance level of *p* < 0.05 was set for all statistical tests to determine statistical significance.

## 3 Results

### 3.1 Baseline characteristics of the study population

This study included 30,347 eligible middle-aged and older participants, comprising 565 PD patients and 29,782 non-PD participants (Table 1). Significant differences were found between PD patients and non-PD participants in sociodemographic variables such as age, gender, education level, and marital status (*p* < 0.05). For example, the incidence of PD was higher in individuals aged 65 and older (53.1%), compared to 46.9% in those aged 45–64 (*p* < 0.05). Gender analysis revealed that the incidence in men was slightly higher than in women (*p* = 0.032). A lower education level was associated with an increased risk of PD, with 72.9% of PD patients having an education level of primary school or below (*p* = 0.003). Marital status was also related to PD risk, with married individuals having a lower risk (*p* = 0.028).

**Table 1:** Baseline characteristics of the study population.

Characteristic	Group	Overall (*n* = 30,347)	Non-PD (*n* = 29,782)	PD (*n* = 565)	χ^2^	*p*-value
Age (%)	45–64 years	21,796 (71.82)	21,531 (72.30)	265 (46.90)	176.662	<0.001
	≥65 years	8,551 (28.18)	8,251 (27.70)	300 (53.10)		
Gender (%)	Female	15,856 (52.25)	15,586 (52.33)	270 (47.79)	4.593	0.032
	Male	14,491 (47.75)	14,196 (47.67)	295 (52.21)		
Education level (%)	Primary school or below	20,235 (66.68)	19,823 (66.56)	412 (72.92)	11.820	0.003
	Middle school	6,577 (21.67)	6,486 (21.78)	91 (16.11)		
	High school or above	3,535 (11.65)	3,473 (11.66)	62 (10.97)		
Marital status (%)	Others	3,901 (12.85)	3,811 (12.8)	90 (15.93)	4.858	0.028
	Married	26,446 (87.15)	25,971 (87.2)	475 (84.07)		
Residence (%)	Rural	18,718 (61.68)	18,354 (61.63)	364 (64.42)	1.835	0.176
	Urban	11,629 (38.32)	11,428 (38.37)	201 (35.58)		
Personal income level (%)	<10,000 RMB	24,085 (79.37)	23,606 (79.26)	479 (84.78)	10.302	0.001
	≥10,000 RMB	6,262 (20.63)	6,176 (20.74)	86 (15.22)		
Smoking (%)	No	12,606 (41.54)	12,344 (41.45)	262 (46.37)	5.536	0.019
	Yes	17,741 (58.46)	17,438 (58.55)	303 (53.63)		
Drinking (%)	No	10,397 (34.26)	10,199 (34.25)	198 (35.04)	0.157	0.692
	Yes	19,950 (34.26)	19,583 (65.75)	367 (64.96)		
Sleep duration (%)	Less than 6 h/day	8,238 (27.15)	8,030 (26.96)	208 (36.81)	27.210	<0.001
	6 h or more/day	22,109 (72.85)	21,752 (73.04)	357 (63.19)		
BMI (%)	BMI < 18.5 kg/m^2^	1,725 (5.68)	1,685 (5.66)	40 (7.08)	2.091	0.148
	BMI ≥ 18.5 kg/m^2^	28,622 (94.32)	28,097 (94.34)	525 (92.92)		
Social activity (%)	No	13,768 (45.37)	13,509 (45.36)	259 (45.84)	0.052	0.820
	Yes	16,579 (54.63)	16,273 (54.64)	306 (54.16)		

BMI, body mass index; PD, Parkinson’s disease; % for categorical variables; *χ*^2^ represents the chi-square test for comparison between the two groups (Non-PD and PD). *p*-values < 0.05 are considered statistically significant.

### 3.2 Association between depression and PD onset risk

The risk of PD incidence (HR) under different levels of depression in four models (Table 2). The results from Model 1, which did not include covariates, show that individuals with depressive symptoms have a 71.7% higher probability of developing PD compared to those without depressive symptoms. In Model 4, after adjusting for sociodemographic variables, lifestyle factors, and health-related variables, the probability of PD incidence for individuals with depressive symptoms increased by 52.9% relative to those without depressive symptoms. Overall, the results from Models 1 to 4 indicate a significant positive correlation between depression and PD, suggesting that individuals with baseline depressive symptoms have a higher risk of developing PD during follow-up.

**Table 2:** Risk of PD onset (HR) in individuals with and without depression.

Model	Risk Status	No-depression	Depression
Model 1	HR	1	1.72
	95%CI	Reference	1.45–2.03
Model 2	HR	1	1.71
	95%CI	Reference	1.44–2.03
Model 3	HR	1	1.65
	95%CI	Reference	1.38–1.96
Model 4	HR	1	1.53
	95%CI	Reference	1.28–1.83

HR, Hazard Ratio; 95% CI, 95% Confidence Interval.

Model 1: Unadjusted model; Model 2: Adjusted for sociodemographic variables; Model 3: Further adjusted for lifestyle variables; Model 4: Fully adjusted model.

We will categorize individuals into two groups based on their lifestyle score (unhealthy lifestyle: total lifestyle score < 4, healthy lifestyle: total lifestyle score ≥ 4), and analyze the association between depression and PD risk separately for each group. Specific results are presented in Supplementary Table S1. The results clearly show that from Model 1 to Model 4, depression is significantly associated with a higher risk of PD in individuals with an unhealthy lifestyle.

We present the HR for the association between depression and various lifestyle factors with the risk of PD (Supplementary Table S2). For smoking and alcohol consumption, individuals with depression exhibited a significantly higher risk of PD compared to those without depression. Additionally, individuals who did not participate in social activities, had shorter sleep duration (≤6 h), or had a body mass index (BMI) < 18.5 kg/m^2^ all demonstrated a higher risk of developing PD.

### 3.3 Dose–response relationship between depression and the incident risk of PD

**Figure 2:**
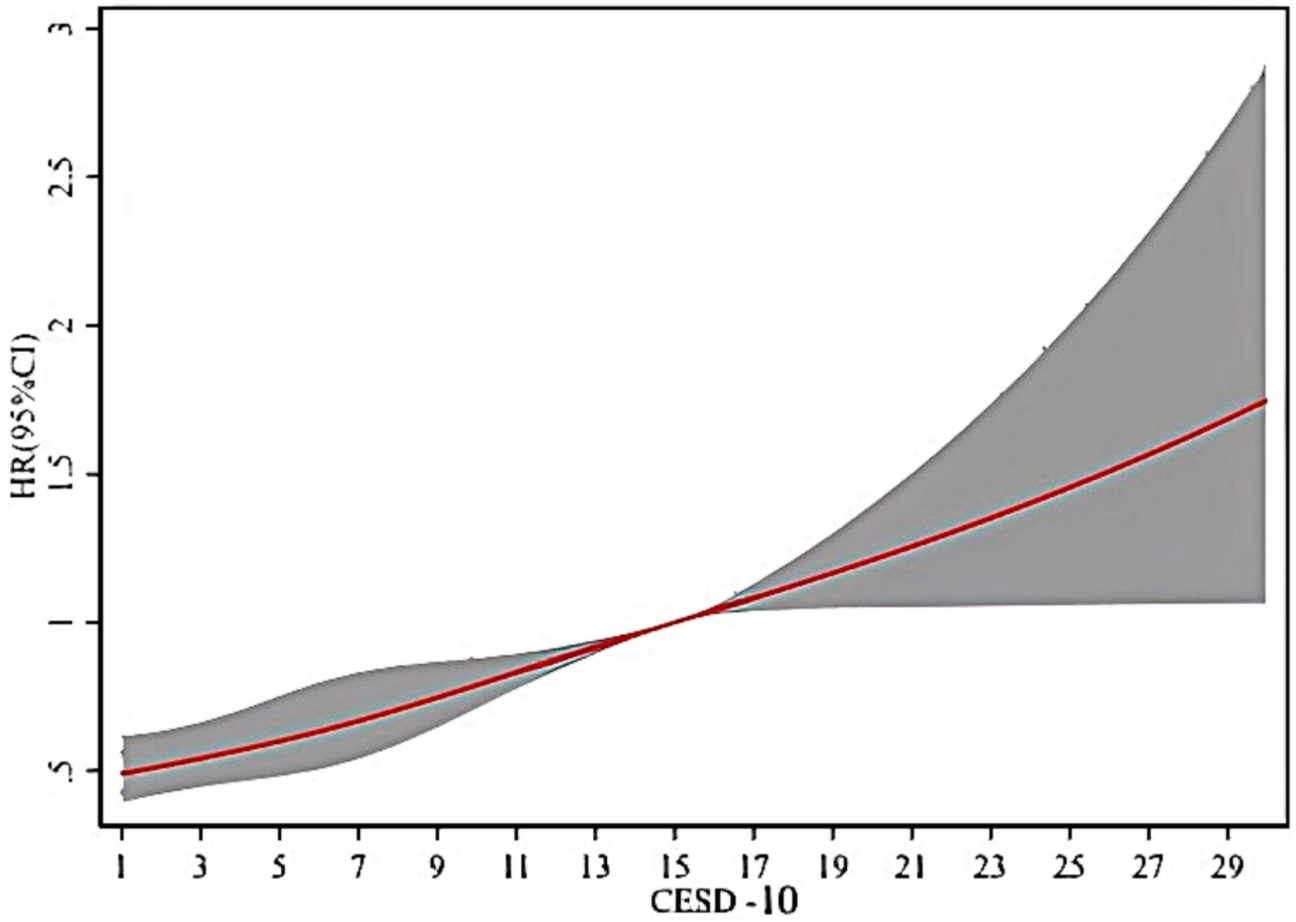
Cubic spline plot of the association between depression severity and PD onset risk.

As shown in Figure 2, it is clear that when the CESD-10 score is 10, the HR is 0.79, (95% CI: 0.70–0.89). There is a significant positive correlation between PD and depression in middle-aged and older adults, with the risk of onset increasing as the CESD-10 score rises.

### 3.4 Relationship between lifestyle and PD risk

The risk of PD incidence (HR) under different classifications in the baseline model (without control variables) (Table 3). The results from the smoking status classification show that no-smoking have a 17.9% lower probability of developing PD compared to smoking. The results from the alcohol consumption classification indicate that non-drinkers have a 3.4% lower probability of developing PD compared to drinkers. The results from the sleep duration classification show that individuals with long sleep duration have a 36.2% lower probability of developing PD compared to those with short sleep duration. The results from the body mass index (BMI) classification indicate that individuals with a normal BMI have a 21.0% lower probability of developing PD compared to those with an abnormal BMI. The results from the social activity participation classification show that individuals who participate in social activities have a 1.9% lower probability of developing PD compared to those who do not. The results from the lifestyle classification indicate that individuals with a healthy lifestyle have a 23.5% lower probability of developing PD compared to those with an unhealthy lifestyle. Therefore, adopting a healthy lifestyle can significantly reduce the risk of PD in individuals with depression, particularly among those who do not smoke, drink little, maintain a normal BMI, and actively participate in social activities, where the effect is most pronounced.

**Table 3:** Risk of PD onset (HR) by different categories.

Category	Group	HR	95%CI
Age	45–64 years	1	Reference
	≥65 years	2.90	2.46–3.42
Gender	Female	1	Reference
	Male	1.20	1.01–1.41
Education level	Primary school or below	1	Reference
	Middle school	0.68	0.54–0.85
	High school or above	0.86	0.66–1.12
Marital status	Others	1	Reference
	Married	0.78	0.62–0.97
Residence	Rural	1	Reference
	Urban	0.89	0.75–1.06
Personal income level	<10,000 RMB	1	Reference
	≥10,000 RMB	0.69	0.55–0.87
Smoking	No	1	Reference
	Yes	0.82	0.70–0.97
Drinking	No	1	Reference
	Yes	0.97	0.81–1.15
Sleep duration	Less than 6 h/day	1	Reference
	6 h or more/day	0.64	0.54–0.76
BMI	BMI < 18.5 kg/m^2^	1	Reference
	BMI ≥ 18.5 kg/m^2^	0.79	0.57–1.09
Social activity	No	1	Reference
	Yes	0.98	0.83–1.16
Lifestyle	Unhealthy	1	Reference
	Healthy	0.77	0.65–0.90

BMI, body mass index; HR, Hazard Ratio; 95% CI, 95% Confidence Interval.

### 3.5 Sensitivity analysis

This study excluded participants with cognitive impairment at baseline to further verify the robustness of the previous conclusions. Table 4 reports the risk of PD onset in participants with and without depression, after excluding those with cognitive impairment. The results from Model 1 to Model 4 show that the risk of developing PD is higher in participants with depression compared to those without depression, indicating a significant positive correlation between depression and PD.

**Table 4:** Results of sensitivity analysis.

Model	Risk Status	No-depression	Depression
Model 1	HR	1	1.75
	95%CI	Reference	1.47–2.07
Model 2	HR	1	1.74
	95%CI	Reference	1.46–2.07
Model 3	HR	1	1.68
	95%CI	Reference	1.40–2.00
Model 4	HR	1	1.56
	95%CI	Reference	1.30–1.87

HR, Hazard Ratio; 95% CI, 95% Confidence Interval.

### 3.6 Relationship between lifestyle and PD risk

**Figure 3:**
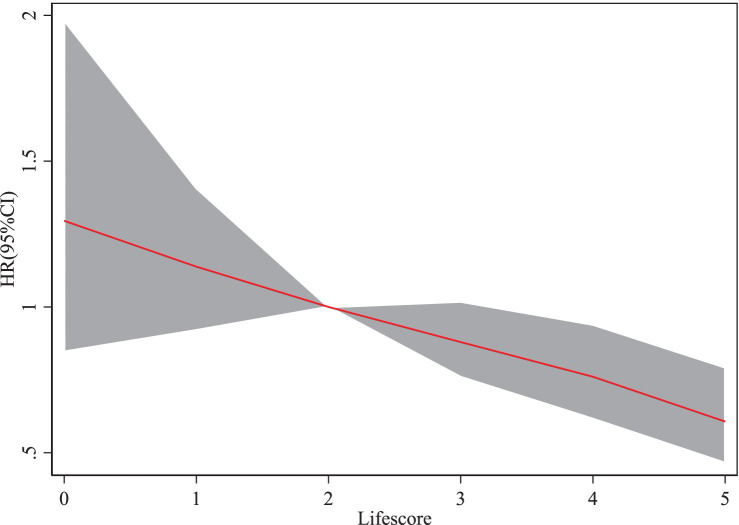
Cubic spline plot of the association between lifestyle and PD onset risk.

When the lifestyle score is 4 (HR = 0.76, 95% CI: 0.62–0.94), the risk of PD (HR) shows a gradual decline as the lifestyle score increases (Figure 3).

## 4 Discussion

Our study results indicate that depression is significantly associated with an increased risk of developing PD. Specifically, individuals with depression have a significantly higher risk of developing PD compared to those without depression. This finding suggests that depression is an important risk factor for PD in middle-aged and older populations. We also found a dose–response relationship between the severity of depression and PD risk; the more severe the depression, the higher the risk of developing PD. Additionally, we observed a significant association between maintaining an unhealthy lifestyle and an increased risk of PD.

The high comorbidity of depression in PD patients is associated with an increased risk of PD, highlighting the importance of addressing depressive symptoms as part of PD risk reduction strategies, with age being a significant factor in this relationship, consistent with previous studies (Cong et al., 2022; Jacobs et al., 2020; Pontone and Mills, 2021). Depression is the most common psychiatric disorder in Parkinson’s patients, with about 35% of PD patients experiencing depression. Depression often appears early in the course of PD and increases with disease progression and age (Broen et al., 2016). In the later stages of the disease, depressive symptoms severely affect the quality of life of patients (Xiao et al., 2024; Weintraub et al., 2022). However, these symptoms are often overlooked, and there is currently a lack of rapid, objective diagnostic biomarkers, making clinical diagnosis challenging (Sun et al., 2024). From a neurobiological perspective, this is directly related to the loss of dopamine nerve terminals and dopamine transporter (DAT) expression in the striatum, which is associated with depressive symptoms in PD (Jacobs et al., 2020; Wang et al., 2025). Additionally, studies based on electroencephalogram (EEG) and event-related potentials (ERPs) have found significant changes in ERPs in Parkinson’s patients with depression, providing new evidence for the neurobiological mechanisms of depression (Sun et al., 2024). There exists a bidirectional relationship between depression and PD, which can be explained through neurobiological mechanisms such as neuroinflammation and dopaminergic dysfunction (Armstrong and Okun, 2020). Dopaminergic denervation, particularly in regions such as the striatum, is closely associated with depressive symptoms in PD patients (Thobois et al., 2010). The complex interplay between PD and depression, involving factors such as disease duration, stage, and comorbidities (Sheikh Hassan et al., 2024), underscores the necessity for timely treatment and prevention. Depression is associated with a longer disease course in PD and is influenced by factors such as gender and education level (Cong et al., 2022; Xu et al., 2025; Hou et al., 2021).

The risk of PD in middle-aged and older adults is related to multiple lifestyle factors. For instance, studies have found that lifestyle factors such as smoking and drinking may be associated with the risk of PD. Research indicates an inverse relationship between smoking and PD, with smokers having a lower risk of developing PD compared to non-smokers (Marinus et al., 2018; Di Biase et al., 2024), which is consistent with our findings. While smoking may reduce the risk of PD, the exact mechanism is unclear, and its protective effect may be offset by other health risks. The impact of drinking on PD remains complex. Although some studies suggest a protective effect of moderate drinking, others have found no significant association, and the findings in this study are consistent with the latter (Jacobs et al., 2020; Domenighetti et al., 2022). Moderate participation in social activities is associated with a lower risk of PD, possibly by enhancing brain health and improving quality of life, thereby contributing to the prevention and management of PD (Ascherio and Schwarzschild, 2016). However, this study did not find a significant association, which may be due to factors such as cultural differences, variations in the types of social activities, or limitations in the methods used to assess them. Sleep disturbances affect about two-thirds of PD patients, and these issues may appear years before motor symptoms develop (Lees et al., 2009). This suggests that sleep disorders could serve as an early warning signal for the future development of PD. The BMI of PD patients is typically lower than the average, and BMI is negatively correlated with the disease duration and severity (Chaudhuri et al., 2006). However, these findings require further research to confirm these associations and explore their underlying mechanisms.

This study has several limitations. Although CHARLS provides a large and representative sample of the Chinese middle-aged and older population, cultural and regional factors may limit the generalizability of the results. The study relied on self-reported PD diagnoses, which may introduce information bias. Despite controlling for various covariates in the analysis, potential confounding factors cannot be entirely excluded. For example, genetic factors play a crucial role in the development of PD, but genetic data were not included in this study. Future research could incorporate genetic information to further explore the complex relationships between depression, lifestyle, and PD risk. Additionally, while temporal precedence was considered, the study remains observational, and therefore, there are limitations in establishing causal inferences.

## 5 Conclusion

This study indicates a significant positive correlation between depression and PD, and a strong association between an unhealthy lifestyle and an increased risk of PD in individuals with depression. These findings provide new perspectives for the prevention and intervention of PD, highlighting the importance of mental health management and lifestyle interventions.

## Data Availability

Publicly available datasets were analyzed in this study. This data can be found here: The data used in this study are publicly released data by CHARLS. Permissions were obtained to access the data used in our research, which were granted by the CHARLS team. The raw data is available on the website (https://charls.pku.edu.cn/en).

## Footnotes

^1^ http://charls.pku.edu.cn/index.htm

## References

[ref1] Alshimemeri S. Alsudais H. Alamri N. K. Alshoumar A. M. Bin Dher S. K. Maashi M. H. (2024). Burden, anxiety, and depression among caregivers of Parkinson’s disease patients. J. Parkinsons Dis. 14, 1495–1505. doi: 10.3233/JPD-240014, PMID: 39365323 PMC11492025

[ref2] Andresen E. M. Malmgren J. A. Carter W. B. Patrick D. L. (1994). Screening for depression in well older adults: evaluation of a short form of the CES-D (center for epidemiologic studies depression scale). Prev. Med. 10, 77–84. 8037935

[ref3] Armstrong M. J. Okun M. S. (2020). Diagnosis and treatment of Parkinson disease: a review. JAMA 323, 548–560. doi: 10.1001/jama.2019.22360, PMID: 32044947

[ref4] Ascherio A. Schwarzschild M. A. (2016). The epidemiology of Parkinson's disease: risk factors and prevention. Lancet Neurol. 15, 1257–1272. doi: 10.1016/S1474-4422(16)30230-7, PMID: 27751556

[ref5] Broen M. P. Narayen N. E. Kuijf M. L. Dissanayaka N. N. Leentjens A. F. (2016). Prevalence of anxiety in Parkinson's disease: a systematic review and meta-analysis. Mov. Disord. 31, 1125–1133. doi: 10.1002/mds.26643, PMID: 27125963

[ref6] Chaudhuri K. R. Healy D. G. Schapira A. H. (2006). Non-motor symptoms of Parkinson's disease: diagnosis and management. Lancet Neurol. 5, 235–245. doi: 10.1016/S1474-4422(06)70373-8, PMID: 16488379

[ref7] Cong S. Xiang C. Zhang S. Zhang T. Wang H. Cong S. (2022). Prevalence and clinical aspects of depression in Parkinson’s disease: a systematic review and meta-analysis of 129 studies. Neurosci. Biobehav. Rev. 141:104749. doi: 10.1016/j.neubiorev.2022.104749, PMID: 35750224

[ref8] Cui S. S. Du J. J. Fu R. Lin Y. Q. Huang P. He Y. C.. (2017). Prevalence and risk factors for depression and anxiety in Chinese patients with Parkinson disease. BMC Geriatr. 17, 270–210. doi: 10.1186/s12877-017-0666-2, PMID: 29166864 PMC5700465

[ref9] Cusso M. E. Donald K. J. Khoo T. K. (2016). The impact of physical activity on non-motor symptoms in Parkinson’s disease: a systematic review. Front. Med. 3:35. doi: 10.3389/fmed.2016.00035, PMID: 27583249 PMC4987718

[ref10] De Lau L. M. Breteler M. M. (2006). Epidemiology of Parkinson's disease. Lancet Neurol. 5, 525–535. doi: 10.1016/S1474-4422(06)70471-9, PMID: 16713924

[ref11] Di Biase L. Pecoraro P. M. Carbone S. P. Alessi F. Di Lazzaro V. (2024). Smoking exposure and Parkinson's disease: a UK brain Bank pathology-validated case-control study. Parkinsonism Relat. Disord. 125:107022. doi: 10.1016/j.parkreldis.2024.107022, PMID: 38865837

[ref12] Domenighetti C. Sugier P. E. Sreelatha A. A. K. Schulte C. Grover S. Mohamed O.. (2022). Mendelian randomisation study of smoking, alcohol, and coffee drinking in relation to Parkinson’s disease. J. Parkinsons Dis. 12, 267–282. doi: 10.3233/JPD-212851, PMID: 34633332 PMC9211765

[ref13] Hou M. Mao X. Hou X. Li K. (2021). Stigma and associated correlates of elderly patients with Parkinson's disease. Front. Psych. 12:708960. doi: 10.3389/fpsyt.2021.708960, PMID: 34335340 PMC8319540

[ref14] Inoue T. Kitagawa M. Tanaka T. Nakagawa S. Koyama T. (2010). Depression and major depressive disorder in patients with Parkinson's disease. Mov. Disord. 25, 44–49. doi: 10.1002/mds.22921, PMID: 20014057

[ref15] Jacobs B. M. Belete D. Bestwick J. Blauwendraat C. Bandres-Ciga S. Heilbron K.. (2020). Parkinson’s disease determinants, prediction and gene-environment interactions in the UK biobank. J. Neurol. Neurosurg. Psychiatry 91, 1046–1054. doi: 10.1136/jnnp-2020-323646, PMID: 32934108 PMC7509524

[ref16] Jankovic J. (2008). Parkinson’s disease: clinical features and diagnosis. J. Neurol. Neurosurg. Psychiatry 79, 368–376. doi: 10.1136/jnnp.2007.131045, PMID: 18344392

[ref17] Jeong W. Kim H. Joo J. H. Jang S. I. Park E. C. (2021). Association between depression and risk of Parkinson's disease in south Korean adults. J. Affect. Disord. 292, 75–80. doi: 10.1016/j.jad.2021.05.038, PMID: 34102551

[ref18] Kalia L. V. Lang A. E. (2015). Parkinson's disease. Lancet 386, 896–912. doi: 10.1016/S0140-6736(14)61393-3, PMID: 25904081

[ref19] Lees A. J. Hardy J. Revesz T. (2009). Parkinson's disease. Lancet 373, 2055–2066. doi: 10.1016/S0140-6736(09)60492-X 19524782

[ref20] Lian T. H. Guo P. Zuo L. J. Hu Y. Yu S. Y. Yu Q. J.. (2019). Tremor-dominant in Parkinson disease: the relevance to iron metabolism and inflammation. Front. Neurosci. 13:255. doi: 10.3389/fnins.2019.00255, PMID: 30971879 PMC6445850

[ref21] Liu Y. Cui J. Cao L. Stubbendorff A. Zhang S. (2024). Association of depression with incident sarcopenia and modified effect from healthy lifestyle: the first longitudinal evidence from the CHARLS. J. Affect. Disord. 344, 373–379. doi: 10.1016/j.jad.2023.10.012, PMID: 37805156

[ref22] Marinus J. Zhu K. Marras C. Aarsland D. Van Hilten J. J. (2018). Risk factors for non-motor symptoms in Parkinson's disease. Lancet Neurol. 17, 559–568. doi: 10.1016/s1474-4422(18)30127-3, PMID: 29699914

[ref23] Paul K. C. Chuang Y. H. Shih I. F. Keener A. Bordelon Y. Bronstein J. M.. (2019). The association between lifestyle factors and Parkinson's disease progression and mortality. Mov. Disord. 34, 58–66. doi: 10.1002/mds.27577, PMID: 30653734 PMC6544143

[ref24] Poewe W. Seppi K. Tanner C. M. Halliday G. M. Brundin P. Volkmann J.. (2017). Parkinson disease. Nat. Rev. Dis. Primers 3, 1–21. doi: 10.1038/nrdp.2017.13 28332488

[ref25] Pontone G. M. Mills K. A. (2021). Optimal treatment of depression and anxiety in Parkinson's disease. Am. J. Geriatr. Psychiatry 29, 530–540. doi: 10.1016/j.jagp.2021.02.037, PMID: 33648830

[ref26] Pringsheim T. Jette N. Frolkis A. Steeves T. D. (2014). The prevalence of Parkinson's disease: a systematic review and meta-analysis. Mov. Disord. 29, 1583–1590. doi: 10.1002/mds.25945, PMID: 24976103

[ref27] Reichmann H. Csoti I. Koschel J. Lorenzl S. Schrader C. Winkler J.. (2022). Life style and Parkinson’s disease. J. Neural Transm. 129, 1235–1245. doi: 10.1007/s00702-022-02509-1, PMID: 35606622 PMC9463300

[ref28] Reijnders J. S. Ehrt U. Weber W. E. Aarsland D. Leentjens A. F. (2008). A systematic review of prevalence studies of depression in Parkinson's disease. Mov. Disord. 23, 183–189. doi: 10.1002/mds.21803 17987654

[ref29] Sheikh Hassan M. Mohamed N. A. Yücel Y. Abdirisak Mohamed Y. Gökgül A. (2024). The prevalence of depressive symptoms in patients with idiopathic Parkinson’s disease: cross-sectional study from Somalia. Int. J. Gen. Med. 17, 5059–5068. doi: 10.2147/IJGM.S493161, PMID: 39526067 PMC11550689

[ref30] Sun Y. Mo Y. Peng C. Li Q. Wang Z. Xue S.. (2024). P1 evoked by facial expression images is enhanced in Parkinson’s disease patients with depressive symptoms. Front. Aging Neurosci. 16:1423875. doi: 10.3389/fnagi.2024.1423875, PMID: 39539459 PMC11557433

[ref31] Thobois S. Ardouin C. Lhommee E. Klinger H. Lagrange C. Xie J.. (2010). Non-motor dopamine withdrawal syndrome after surgery for Parkinson's disease: predictors and underlying mesolimbic denervation. Brain 133, 1111–1127. doi: 10.1093/brain/awq032, PMID: 20237128

[ref32] Tolosa E. Garrido A. Scholz S. W. Poewe W. (2021). Challenges in the diagnosis of Parkinson's disease. Lancet Neurol. 20, 385–397. doi: 10.1016/S1474-4422(21)00030-2, PMID: 33894193 PMC8185633

[ref33] Van Den Eeden S. K. Tanner C. M. Bernstein A. L. Fross R. D. Leimpeter A. Bloch D. A.. (2003). Incidence of Parkinson’s disease: variation by age, gender, and race/ethnicity. Am. J. Epidemiol. 157, 1015–1022. doi: 10.1093/aje/kwg068, PMID: 12777365

[ref34] Wang P. H. Chang Y. P. Chien C. F. Huang P. (2025). Differential striatal dopamine binding in Parkinson’s disease with and without REM sleep behavior disorder: a Tc-99 m TRODAT-1 SPECT study. Geroscience 47, 2581–2591. doi: 10.1007/s11357-024-01500-w, PMID: 39775602 PMC11978563

[ref35] Wang S. Mao S. Xiang D. Fang C. (2018). Association between depression and the subsequent risk of Parkinson's disease: a meta-analysis. Prog. Neuro-Psychopharmacol. Biol. Psychiatry 86, 186–192. doi: 10.1016/j.pnpbp.2018.05.025, PMID: 29859854

[ref36] Weintraub D. Aarsland D. Chaudhuri K. R. Dobkin R. D. Leentjens A. F. Rodriguez-Violante M.. (2022). The neuropsychiatry of Parkinson's disease: advances and challenges. Lancet Neurol. 21, 89–102. doi: 10.1016/S1474-4422(21)00330-6, PMID: 34942142 PMC8800169

[ref37] Xiao H. Ren Y. Yang H. Wang Z. Li Z. Song Y.. (2024). Acupuncture for early Parkinson’s disease with mild to moderate depression: a randomized controlled trial protocol with functional MRI. Front. Neurol. 15:1457787. doi: 10.3389/fneur.2024.1457787, PMID: 39430584 PMC11486739

[ref38] Xu Y. Chen D. Dong M. Zhang Y. Yu H. Han Y. (2025). Bidirectional relationship between depression and activities of daily living and longitudinal mediation of cognitive function in patients with Parkinson's disease. Front. Aging Neurosci. 17:1513373. doi: 10.3389/fnagi.2025.1513373, PMID: 40013091 PMC11861111

[ref39] Zhou L. Ma X. Wang W. (2021). Relationship between cognitive performance and depressive symptoms in Chinese older adults: the China health and retirement longitudinal study (CHARLS). J. Affect. Disord. 281, 454–458. doi: 10.1016/j.jad.2020.12.059, PMID: 33360747

