# Supplementary material for: The association between the incident risk of Parkinson’s disease and depression in middle-aged and older adults, and the moderating role of lifestyle: evidence from the CHARLS
Source: Front Psychol. 2025 Jun 18;16:1590931. doi: 10.3389/fpsyg.2025.1590931 (PMC12213753; doi:10.3389/fpsyg.2025.1590931)
Supplement: Supplementary file 1 [file Data_Sheet_1.pdf]

We will categorize individuals into two groups based on their lifestyle score (unhealthy lifestyle: total lifestyle score < 4, healthy lifestyle: total lifestyle score ≥ 4), and analyze the association between depression and PD risk separately for each group. Specific results are presented in **Supplementary Table 1**. The results clearly show that from Model 1 to Model 4, depression is significantly associated with a higher risk of PD in individuals with an unhealthy lifestyle.

Supplementary Table 1: Depression and PD incidence risk (HR) by lifestyle

Lifestyle	Depression	Model 1	Model 2	Model 3	Model 4
Unhealthy	No-depression	Ref.	Ref.	Ref.	Ref.
	depression	2.35 (1.84-3.00)	2.32 (1.80-3.00)	2.29 (1.77- 2.97)	2.10 (1.61- 2.74)
Healthy	No-depression	Ref.	Ref.	Ref.	Ref.
	depression	1.29 (0.97-1.71)	1.32 (0.99-1.77)	1.28 (0.95- 1.72)	1.22 (0.90-1.65)
