# Supplementary material for: The association between the incident risk of Parkinson’s disease and depression in middle-aged and older adults, and the moderating role of lifestyle: evidence from the CHARLS
Source: Front Psychol. 2025 Jun 18;16:1590931. doi: 10.3389/fpsyg.2025.1590931 (PMC12213753; doi:10.3389/fpsyg.2025.1590931)
Supplement: Supplementary file 2 [file Data_Sheet_2.pdf]

Supplementary Table 2: HR for the risk of PD in relation to depression and five different lifestyle factors

Lifestyle	Depression	Model 1	Model 2	Model 3	Model 4
No-smoking	No	Ref.	Ref.	Ref.	Ref.
	Yes	1.67 (1.30-2.16)	1.61 (1.24-2.08)	1.59 (1.22-2.08)	1.43 (1.09-1.87)
Smoking	No	Ref.	Ref.	Ref.	Ref.
	Yes	1.83 (1.46-2.29)	1.79 (1.42-2.26)	1.68 (1.33-2.13)	1.61 (1.27-2.05)
No-drinking	No	Ref.	Ref.	Ref.	Ref.
	Yes	1.66 (1.23-2.23)	1.57 (1.15-2.13)	1.53 (1.12-2.09)	1.35 (0.98-1.86)
Drinking	No	Ref.	Ref.	Ref.	Ref.
	Yes	1.77 (1.44-2.18)	1.78 (1.44-2.19)	1.70 (1.37-2.10)	1.61 (1.30-2.01)
Participating in Social Activities	No	Ref.	Ref.	Ref.	Ref.
	Yes	1.61 (1.26-2.06)	1.59 (1.24-2.04)	1.53 (1.19-1.97)	1.42 (1.09-1.83)
Not participating in Social Activities	No	Ref.	Ref.	Ref.	Ref.
	Yes	1.82 (1.45-2.30)	1.84 (1.45-2.34)	1.77 (1.39-2.27)	1.65 (1.28-2.12)
Sleep Duration >6 hours	No	Ref.	Ref.	Ref.	Ref.
	Yes	1.43 (1.09-1.88)	1.49 (1.13-1.96)	1.49 (1.13-1.96)	1.45 (1.09-1.93)
Sleep Duration ≤6 hours	No	Ref.	Ref.	Ref.	Ref.
	Yes	1.71 (1.38-2.13)	1.73 (1.38-2.17)	1.77 (1.41-2.22)	1.60 (1.27-2.02)
BMI≥18.5 kg/m²	No	Ref.	Ref.	Ref.	Ref.
	Yes	0.96 (0.51- 1.82)	0.99 (0.52-1.93)	0.92 (0.48- 1.79)	0.86 (0.43- 1.71)
BMI<18.5 kg/m²	No	Ref.	Ref.	Ref.	Ref.
	Yes	1.79 (1.50-2.13)	1.78 (1.49-2.13)	1.72 (1.43- 2.06)	1.60 (1.32- 1.92)
